# Choice on the menu in residential aged care: An underrated tool for maintaining resident autonomy

**DOI:** 10.1111/1747-0080.70002

**Published:** 2025-02-25

**Authors:** Mikaela Wheeler, Karen L. Abbey, Sandra M. Capra

**Affiliations:** ^1^ Faculty of Medicine, School of Public Health The University of Queensland Herston QLD Australia; ^2^ Faculty of Health and Behavioural Science, School of Human Movement and Nutrition Science The University of Queensland St Lucia QLD Australia

**Keywords:** aged care, autonomy, foodservice, long term care, older adults, qualitative research

## Abstract

**Aims:**

This qualitative study aimed to explore residents' experiences of autonomy in relation to their meals pre and post‐intervention, which implemented a restaurant‐style dining service and increased the number of meal choices available in one residential aged care home.

**Methods:**

Participants completed semi‐structured interviews pre‐ and post‐intervention. Interviews were transcribed and analysed using reflexive thematic analysis. Adopting an interpretivist approach, the researchers prioritised participants' subjective experiences and engaged collaboratively in reflexive practices to examine their positionality while developing themes.

**Results:**

Twenty participants completed pre‐interviews and eighteen completed post‐interviews. Themes developed from pre‐intervention interviews included ‘Autonomy in residential aged care homes: Ideal versus reality’, ‘Values and expectations of residential aged care’ and ‘Disempowerment within residential aged care systems’. Themes from post intervention interviews included ‘Reclaiming Autonomy’ ‘System design to support autonomy and Autonomy beyond meal choice’.

**Conclusion:**

Residents in residential aged care encounter institutional factors that undermine their sense of autonomy. However, foodservice systems have the potential to enhance choice and autonomy and positively impact residents.

## 1 INTRODUCTION

Moving into a residential aged care home marks a significant change in a person's life, and residents have reported feelings of helplessness, lack of optimism, abandonment, vulnerability and some anger at the injustice.^1^, ^2^ A key concern for residents is losing control, independence, freedom and lack of continuity with their past life.^3^, ^4^ Being autonomous refers to the ability to live according to an individual's beliefs, preferences, motives and promotes good health and quality of life.^5^, ^6^ Institutional factors such as sharing a bathroom, routines and regulations for meals, waking and bathing, and lack of meaningful in‐house activities all contribute to the loss of autonomy. Therefore, residential aged care homes must be designed to support residents to be autonomous in their day‐to‐day lives.

Food and mealtimes are areas where residents want to express their autonomy. In the community, individuals make decisions about food based on preferences, which have been shaped by a lifetime of experiences.^7^, ^8^ Being able to choose foods that align with these preferences is a sign of normality and a continuation of self‐identity.^9^ However, due to the organisational design of residential aged care homes, there is a shift in the roles between residents and administrators. Residents, who were once experts in their own food and preferences, become recipients. At the same time, administrators assume the position of expert and decision maker, setting the menus and deciding the type and quantity of foods that will be provided.^10^ This loss of autonomy impacts both the physical and mental health of residents.^11^

With up to 40% of residents either malnourished or at risk of malnutrition in residential aged care, interventions are required to improve residents' food intake and support their nutritional status.^12^ Foodservice solutions, such as increasing choice, can be utilised to increase foodservice satisfaction and intake.^13^, ^14^ By increasing the choice of menus, homes can increase food variety and better support residents in choosing foods that align with their needs and preferences.^15^ Beyond nutritional status, providing opportunities to make decisions supports residents to affirm their identity through small but meaningful choices.^16^

While the importance of providing choice is well known, current foodservice systems in residential aged care homes provide limited choice in practice, restricting opportunities for residents to be autonomous.^17^, ^18^, ^19^, ^20^ The Royal Commission into Aged Care Quality and Safety also highlighted important concerns with current food and nutrition in residential aged care, stressing the immediate need to improve the food provided to meet the nutritional needs of residents.^21^ In order to address these concerns and improve resident autonomy, new foodservice models are required. This paper reports the qualitative results of a larger mixed‐method trial that modified a foodservice system to increase the number of choices provided to residents in one residential aged care home. Specifically, this study aimed to explore residents' experiences of autonomy regarding their meals, pre‐ and post‐intervention. Quantitative results for this study have been reported elsewhere.^22^

## 2 METHODS

A pre‐post mixed method, pragmatic action research study was completed in one residential aged care home in Queensland, Australia, during 2020–2021. Ethical approval was granted by the University of Queensland Human Research Ethics Committee (2020002082). This study has been reported following the Consolidated Criteria for Reporting Qualitative Research guidelines.^23^

The study was completed in a privately owned home (36 beds), which provided care to residents with varying needs, including those with a diagnosis of dementia. The average length of stay was 5.9 ± 3.48 years, with an average resident age of 75 ± 15.48 years.

Semi‐structured interviews were completed with residents pre‐ and post‐intervention as part of the intervention design process.^24^ Semi‐structured interview guides (Data S1) were developed before conducting the interviews. Pre‐intervention semi‐structured interviews were completed with individual participants. They were designed to gain a deeper understanding of how choice was conceptualised, the expectations of choice in residential aged care and guide intervention development. A second semi‐structured interview was completed post‐intervention to explore the impact of the system on residents.

Before the intervention, the home prepared all meals on‐site and provided the option of a hot meal or salad and dessert at lunch, soup, a small hot meal or sandwich and fruit at dinner. The intervention altered the foodservice system to increase the number of choices provided to residents at lunch and dinner over a 9‐week period. To increase the number of hot options on the menu without significantly increasing staffing requirements within the foodservice, ready‐made meal products were introduced into the menu. The new menu included six ready‐made meal options, one cook‐serve option and two cold options (sandwich or salad). The cook serve option was prepared by the chef onsite. The ready‐made meal options changed every 3 weeks, and the cook‐serve meal was changed daily.

Researchers provided an overview of the study and study documents to a designated registered nurse in the facility who recruited residents over a 2‐week period during face‐to‐face interactions. Further advertisement of the study was also published in the home's newsletter and at resident meetings. Given the small size of the home, all residents were invited to participate if they could participate in an interview. For residents with a diagnosis of dementia, the registered nurse reviewed the interview guide and used clinical judgement to decide which residents might be able to participate. If a resident could participate in an interview but did not provide written consent, a relative, guardian, person or organisation authorised by law would be able to provide consent for that resident. To ensure verbal consent was obtained from the resident to commence the interview, researchers asked participants if they would like to complete the interview. If a resident expressed they did not wish to be interviewed or showed any behaviours that signalled distress or discomfort, researchers assumed that the participant was withdrawing their consent and did not continue with the interview.

The development of interview guides was informed by current literature and the Australian Aged Care Quality and Safety Standards.^25^ Interview questions are provided as Data S1. Pilot interviews were conducted in person by the researcher with three residents to confirm the appropriateness of questions and length of the interview. The pilot interviews also enabled the interviewer to improve their interviewing skills. All transcripts from pilot interviews were included in the final data analysis as minimal changes were made to the interview guide.

Interviews were conducted in person in residents' rooms. Participants were made aware of the purpose of the study and the background of the female researcher (PhD candidate, dietitian, not known to any participant previously). The interviewer and the participant were present for interviews, and no repeat interviews were completed. Interviews were voice recorded using an MP3 audio recorder and transcribed verbatim by the candidate and a research assistant. Field notes about issues, processes, and impressions were recorded during the interview and used in the qualitative analysis. Participants were offered the option to receive a copy of their interview transcript to provide feedback and ensure accuracy; however, no participant elected to receive a copy.

Given the qualitative methods employed in this research, the authors acknowledge their positionality and how this has shaped the research process and the interpretation of results.^26^ Grounded in an interpretivist framework, the researchers prioritised the subjective experiences of participants, while acknowledging their position as outsiders, who do not hold lived experience of the phenomenon being explored.^27^, ^28^ As outsiders, researchers may have provided a neutral and safe space for participants to engage in the research. Indeed, the confidentiality and separation of researchers from the residential aged care home and its staff was confirmed by participants as they shared their experiences. The researchers also acknowledge the power differentials that exist between the researchers and those being researched, which may impact the engagement of participant in the research process.^29^ The research teams' own perspectives and values, particularly as dietitians who work with older adults, shape the interpretation of the data.

Transcripts were analysed using reflexive thematic analysis to identify patterns of meaning across interviews and construct themes. A predominantly inductive approach was used to analyse transcripts; however, a level of deductive analysis was used due to the use of semi‐structured interview guides and focus on particular concepts in order to answer the research questions. The six phases of thematic analysis identified by Braun and Clarke,^30^ (1) familiarisation of the data, (2) generating initial codes, (3) generating themes, (4) reviewing potential themes, (5) defining and naming themes, (6) producing the report, were used to complete the analysis. Recordings of interviews were listened to in full before reading transcripts in order to familiarise the researcher with the data. Transcripts were then read multiple times to further deepen the researchers' familiarity and understanding of the data. Using NVIVO software (version 1.6.1), brief, initial codes were developed and reviewed to understand patterns of meaning across transcripts and construct themes and sub‐themes by one researcher. The construction of themes involved the sorting of codes into overarching concepts. A collaborative and reflexive approach was adopted, with a second researcher reviewing the transcripts and contributing to theme development by examining illustrative quotations identified by the first author. Both authors met multiple times to discuss and revise themes as they were developed.

## 3 RESULTS

**FIGURE 1:**
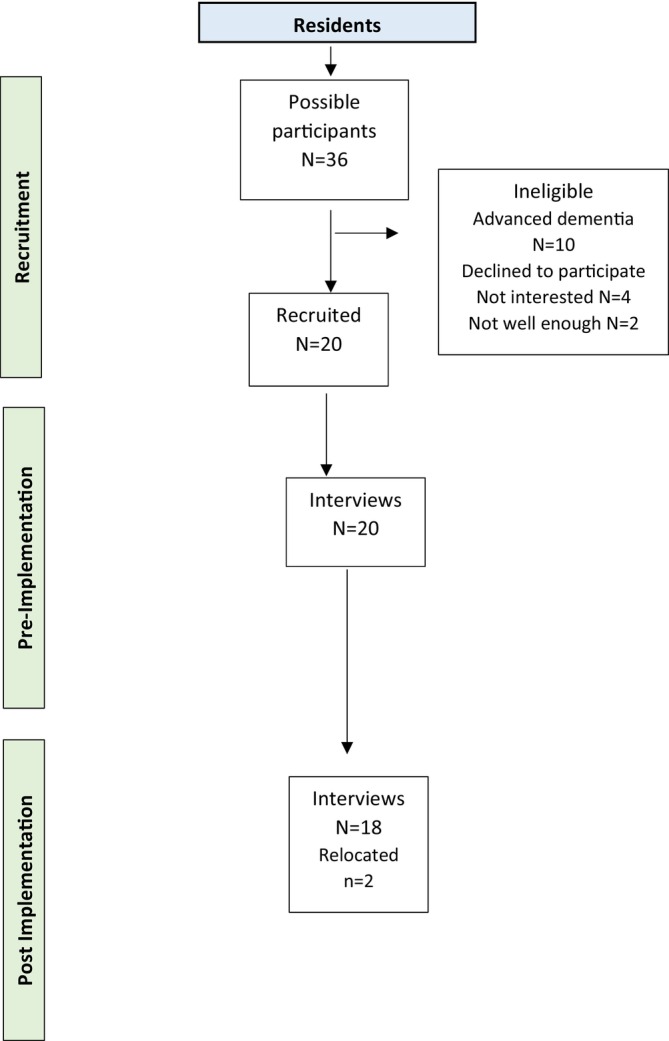
Participant recruitment to pre‐implementation and post‐implementation interviews

Of the 36 residents who lived in the home, 10 were ineligible to participate due to advanced dementia and an inability to communicate verbally. Six residents declined to participate due to not being interested (*n* = 4) or not being well enough (*n* = 2). A total of 20 residents completed a pre‐implementation interview and 18 completed post‐implementation interviews. Two residents did not complete post‐interviews due to relocation to another home (Figure 1).

Sixty percent of participants were female (*n* = 60%). There was a large spread in participant ages, varying from 55 to 95+ years, with the majority of residents (*n* = 47%) aged between 65 and 74 years old. The majority of residents (*n* = 63%) had lived at the home for longer than 3 years (Table 1). The average duration of pre‐intervention interviews was 21 min (range 7–38) and post‐intervention interviews were 18 min (range 5–35).

**TABLE 1:** Participant characteristics.

Characteristic	*N* (%)
Gender	
Female	12 (60)
Age	
55–64 years	2 (10)
65–74 years	9 (45)
75–84 years	4 (20)
85–94 years	4 (20)
95+ years	1 (5)
Length of stay	
<3 years	7 (37)
>3 years	12 (63)
Appetite^a^	
About normal	16 (84)
Better than normal	2 (11)
Worse than normal	1 (5)
Health^a^	
Excellent	0
Very good	1 (5)
Good	9 (47)
Fair	7 (37)
Poor	2 (11)

^a^ Self‐rated as measured by the Resident Foodservice Satisfaction Questionnaire.

Researchers developed three major themes and three sub‐themes in the pre‐intervention interviews and three major themes in the post‐intervention interviews.

The first theme, ‘Autonomy in residential aged care homes: Ideal versus reality’, was developed from pre‐intervention interviews. Participants viewed residential aged care as their home and, as such, expected opportunities to make decisions about food and mealtimes to be provided. The unique and individual preferences held by residents also increased the importance of being able to make decisions and support individualism in a group setting. By making choices, residents felt less institutionalised and reported a greater sense of autonomy, which was identified as necessary. Making decisions about everyday life actions, such as what to eat, were opportunities to reaffirm their autonomy.

You must have a choice. Life is about choices. The decisions you make. It's not being told to eat a salad sandwich when you don't want a salad sandwich. Eat this, when you don't like that.

(#16)

While participants clearly expressed the importance of autonomy in residential aged care, their personal experiences did not support autonomy. Participants stated that they had little to no choice within the current system, with the menu of one hot meal or a salad or sandwich seen as an inadequate level of choice. By having limited meal options to choose from, participants felt they were told what to eat, and as a result, their sense of autonomy was negatively impacted.

You don't always get your choice. What you choose is what they tell you to choose. That's what you're going to eat.

(#12)

The opportunities to make choices were also seen as unequal among residents, with those who were more physically able to advocate for themselves receiving greater choices. This was of concern for participants, feeling this was unjust and further reduced the autonomy of the most vulnerable residents.

I probably get more choice than most because I can go to the kitchen and ask for some eggs, and I boil them myself for supper. Otherwise, you don't get a choice.

(#02)

The second theme developed was ‘Values and expectations of residential aged care’. Participants had mixed expectations of the home and its foodservice, which were largely informed by participants' life experiences and the costs associated with living in residential aged care homes. Those who had worked in hospitality reflected on the challenges of catering for large groups of people and discussed the importance of having realistic expectations. For others, their expectations were informed by negative experiences of living in other homes and by comparison, were satisfied with their current home.

I think for everybody here to have options, it would be terribly hard. I have done that job of cooking for many people.

(#19)

The costs associated with living in a residential aged care home also informed the expectations of some participants, with perceived high costs increasing the expectation for good quality meals. For some participants, the quality of the food provided did not meet their expectations, given their financial investment. This led to a level of dissatisfaction, and some participants felt let down by what was provided.

A lot goes into these places, you pay your board, it covers a lot of things. Surely there's enough left over for a bit of food.

(#01)

Participants valued good quality foodservices, and this encompassed well‐trained staff, the use of good quality ingredients and sufficient meal variety. The staff played a pivotal role both in the kitchen and dining room in providing an enjoyable mealtime. Within the kitchen, some staff lacked skills in food preparation, which impacted the production of quality meals. Participants reported a lack of enjoyment of these meals which then impacted their overall mealtime experience. In the dining room, the reported disorganisation of staff to deliver meals and support the mealtime negatively impacted the mealtime experience.

It seems to be when the other ones are in the kitchen that things might not be quite up to the same standard.

(#17)

The use of good quality ingredients was highly valued by participants and played an important role in producing good quality meals. Participants identified that lower‐cost ingredients were used as substitutes, and this lowered the meal quality. Participants also spoke of limited variety within the menu, leading to a monotonous routine. Repetition was reported in meal ingredients, such as using leftover ingredients across multiple meals in subsequent days and in the menu pattern.

Most of the time what we get in the evenings is the same as what we got yesterday and the day before.

(#08)

The third theme developed was ‘Disempowerment within residential aged care systems’. The sub‐theme ‘Lack of meaningful input’ was developed within the larger theme. Participants reported a lack of input into the running of the home, with their current involvement seemingly superficial. Receiving and recording feedback was perceived as something the home had to do for regulation purposes, and this drove the practice of resident meetings and surveys rather than facilitating actual change. Participants also felt changes were not made in response to their feedback. This resulted in participants feeling disempowered in their ability to influence what happened in their homes.

They might listen, but they don't do anything about it. It goes in one ear and out the other.

(#09)

The second sub‐theme, ‘Fear of retribution’ was also developed within the overarching theme. Participants also discussed their fear of retribution from management if they provided negative feedback. One participant discussed getting ‘*into trouble*’ *(#08)* if they spoke out. As a result, there was a perception that this negatively impacted other residents from providing feedback. While some participants implied negative repercussions, others more explicitly discussed actions they believed were taken against them due to providing negative feedback.

I don't find it to do me any good to complain.

(#19)

The sub‐theme, ‘Authoritative approach to management’ was developed within this broader theme. Within the home, participants spoke of the control management exerted over staff and residents and the lack of control both groups had to make changes to the home. Residents perceived a lack of will on behalf of management which restricted changes from being introduced. Management was also seen as intervening in areas of the home and restricting staff capabilities. In relation to foodservice, some participants felt staff were capable of delivering a higher quality service but were restricted from management to do so.

They know that management isn't going to change it. That's where the problem is, in that office.

(#02)

From post‐intervention interviews, themes developed included ‘Reclaiming autonomy’, ‘System design to support autonomy’ and ‘Autonomy beyond meal choice’. The first theme developed was ‘Reclaiming Autonomy’. The increased number of options provided by the new system offered greater opportunities for participants to make choices about the foods they consumed. These opportunities were highly valued by participants who enjoyed being able to make decisions based on their individual preferences.

Well, you have a choice now, rather than having one selection. If you don't like it, the number of choices allows you to change your mind. Whereas having only one selection, you might not want it.

(#07)

Compared to pre‐intervention interviews, participants' discussions focused more strongly on their own needs and preferences. While still understanding the challenges of providing meals in aged care, participants reflected on their own individuality and the need for systems to support this. Participants discussed that by making choices about their meals each day, they had reclaimed some control over their daily lives. The new system also reduced the number of occasions where staff chose meals for residents if they were absent for meal orders during the day prior, significantly impacting residents' opportunities to be autonomous.

Half the time I might not have been there and that's how I used to get lasagne. If I had the option then I would say that I can't eat that, I'd rather have a sandwich.

(#10)

The second theme developed was ‘System design to support autonomy’ The design of foodservice systems can act as an enabler of resident autonomy through careful planning and design. The new system reflected a restaurant style, which allowed residents to peruse a menu and select their meal at mealtime. This altered the interactions within the dining room, with participants engaging in discussions about their meal selections with both staff and other residents.

It pleases us, it's like going to a restaurant, you look down the list and think, ‘I'll have a bit of that’. It's all round good.

(#14)

Restaurant‐style dining also allowed participants to make decisions about their meals at the mealtime instead of the day prior. This enabled residents to make decisions based on their current preferences, and having this opportunity was considered important. Selecting meals the day prior, which was standard practice, was seen as problematic as participants felt ‘stuck’ with their meal selection despite their preferences changing.

You might not feel like eating it anymore, so what do you do? You have to eat it because you ordered it the day before.

(#16)

An unintended benefit of the new system was the removal of restrictions which had previously limited residents' ability to make meal requests. Participants spoke of restrictions, such as being unable to add ingredients to meals, preventing them from customising their meal selections. The new system provided a new opportunity to review the foodservice and ensure residents felt supported in making personalised requests.

They stopped me ordering cheese and onion and I ordered it the other night, and …I got it and it was good. I enjoyed it… Before you couldn't even order tomato in your sandwich and now you can.

(#09)

The third theme developed was ‘Autonomy beyond meal choice’. Participants reported improvements in the opportunities to make choices; however, the maintenance of previous work patterns, such as only serving dessert once all residents at the table had finished their meal, continued to negatively impact their autonomy mealtimes.

They used to say that everybody at the table needs to finish before the sweets are delivered. So, they do that halfway now, sometimes they deliver the sweets and sometimes they don't. I prefer to have it when I'm finished.

(#11)

Participants also wanted further opportunities to be autonomous, particularly around simple tasks such as making cereal or tea and coffee. Participants viewed these opportunities to not only increase their autonomy but also reduce the pressure on staff in the dining room. For participants, autonomy was not only exercised through meal choices but also in more minor decisions around food and meals.

## 4 DISCUSSION

This study has provided insight into how menus and foodservice systems can impact resident autonomy and satisfaction in residential aged care. Through the analytical process, the researchers developed three major themes from pre‐intervention interviews: *Autonomy in* residential aged care: *Ideal* versus *reality, Values and expectations of* residential aged care *and Disempowerment within* residential aged care *systems*. Within the theme of *Disempowerment within residential aged care systems*, three sub‐themes were developed: *lack of meaningful input, fear of retribution* and *authoritative approach to management*. From post‐intervention interviews, researchers developed three additional major themes: *Reclaiming Autonomy, System design to support autonomy* and *Autonomy beyond meal choice*.

In this study, participants discussed a lack of choice and their dissatisfaction with the design of the system, which limited their opportunities to make decisions about their meals. Dissatisfaction with the level of choice provided in residential aged care more broadly is well documented and has been observed over many decades.^18^, ^31^, ^32^, ^33^, ^34^ Wang et al.^35^ discussed limited food choices available for residents and how this contributed to a monotonous routine. Despite consistent evidence demonstrating the importance of choice for residents and its positive impact on resident satisfaction, quality of life and weight, low satisfaction with choice remains.^36^, ^37^ The importance of choice has also been highlighted in the draft of the new strengthened Australian Aged Care Quality Standards.^38^ Standard six, which sets out obligations for food and dining, requires menus to be designed in partnership with older people, and for each meal, older people are enabled to exercise choice about what, when, where, and how they eat and drink. While this standard successfully brings choice to the forefront of service delivery, there are still challenges in implementing choice due to its ambiguity and lack of available evidence to provide practical guidance for homes to increase choice.^39^ Rigid structures and routines in residential aged care are known to create institutional environments which negatively impact resident autonomy.^40^ Enabling resident choice, including the types of foods consumed, portion size and position to sit in the dining room, are opportunities for residential aged care homes to deliver person‐centred care; however, these moments can be missed due to the system's design and staff organisation.^32^ In this study, residents felt that a more home‐like environment could be created by providing choice. By making decisions about mealtimes, residents perceive that they are taking care of their own needs and exerting autonomy.^33^ The positive impacts of this system were seen in participants' discussion of the enjoyment of making choices that aligned with their personal preferences. Individualistic discussion was more apparent in post‐intervention interviews, with participants reflecting on their preferences and how they differed from other residents, which could be accommodated under the new system. Systems that enable residents' choice, such as the intervention in this study, are needed to provide opportunities for residents to make choices and support their autonomy.

There are limited studies that have increased choice for residents in residential aged care and utilised qualitative methods to understand the resident experience. However, Kenkmann and Hooper^41^ implemented an in‐house restaurant, increasing the number of choices available to residents across mealtimes. Like this study, residents discussed the positive impacts of the new system, including improved quality and variety within the menu. The mealtime also became more social and was a time to greet and connect with fellow residents. Interestingly, residents who required greater support from staff, such as those with physical or cognitive impediments, could not make full use of the system as it was highly dependent on staff availability.^41^ Within the current study, participants highlighted the unfairness of some residents not receiving the same level of choice due to their inability to advocate for themselves. This highlights the importance of designing systems that consider and address the varying needs of residents.

Pre‐implementation interviews highlighted concerning themes of a system that disempowered residents. Participants reported they had little input into the running of the home, and any feedback provided was likely ignored as they failed to see changes occur visually. Concerningly, residents saw the gathering of feedback as a superficial task used to meet accreditation requirements but not to make changes to the home. This practice of exclusion from decision‐making has been reported previously as residents spoke of their lack of input into menus, feeling their opinions went unheard and, as a result, were isolated from all food‐related decisions.^42^

Participants also discussed their own or the apprehension of other residents to make complaints for fear of retribution. In the management‐resident relationship, residents perceived management to hold the majority of the power, which led to an unbalanced power dynamic and an inability to speak up against actions within the home. Fear of retribution exists within residential aged care, with residents navigating the fine line between ‘complaining and remaining quiet’ and the concern that too many complaints may prompt management to close the facility.^43^ Submissions to the Royal Commission into Aged Care Quality and Safety in Australia reported that older people, their families and aged care workers may be reluctant to raise concerns due to a fear of retribution.^21^ This is despite aged care standards requiring residents, family, friends and carers to be encouraged and supported to provide feedback and make complaints.^25^ Perceived power imbalances between residents, staff and management of homes are a significant barrier to resident engagement and, more broadly, resident autonomy. In order to support resident autonomy, residential aged care home environments must include residents directly in all decisions and empower residents to participate in discussions about the operation of their homes.

Improving the oral intake of residents is considered critical to reducing the risk of unintentional weight loss and malnutrition in residential aged care.^44^ By increasing the number of choices available to residents, homes can promote autonomy and support residents in consuming foods that align with their preferences and meet their nutritional needs. By involving residents directly in the mealtime and shifting decision‐making, residents can be empowered to make decisions about their nutrition. This study provides evidence that increasing choice can positively impact residents.

The limitations of this study include the atypical participant population and potential interviewer bias. Participants in this study were somewhat atypical of residents in residential aged care in Australia, as they were younger and had lived in the home longer than national averages. Typically, the median length of stay for permanent residential aged care is 20.5 months, with the majority of residents (*n* = 58%) aged 85 and over.^45^ This may mean the participants in this study were better able to articulate their preferences and participate in system design more than the average resident in residential aged care. While residents with a diagnosis of dementia were encouraged to participate, specific interview guides designed for residents with advanced dementia were not developed due to the limited scope of the study. Given the high percentage of residents who have a diagnosis dementia in residential aged care homes, further work is needed to understand how the system impacts these residents and how they can be further supported to make choices about their mealtimes. There is also bias introduced through the location of researchers in the home and data collection of resident interviews. The act of researchers participating in the intervention design process may also introduce a bias for residents to provide more positive feedback due to the development of a working relationship with researchers during the design process.

This study has demonstrated that increasing the flexibility of foodservices to provide greater choice has a positive impact on residents and their reported autonomy. The individuality of participants was evident and the need for services to meet individual preferences was apparent. Current foodservice systems and the design of residential aged care homes can result in the disempowerment of residents and further work needs to be done to ensure residents are empowered to play active roles in their homes. While system change can be difficult and present many challenges, foodservices can be designed to increase choice and empower residents to have an active role in the mealtime and make decisions based on their preferences.

## AUTHOR CONTRIBUTIONS

Conceptualisation, methodology, Writing – review and editing: MW, KA and SC. Data analysis: MW and SC. Writing – original draft preparation: MW. Supervision: KA and SC. All authors have read and agreed to the final version of the manuscript submitted for publication. The authors acknowledge the staff of the residential aged care home and the research assistants who assisted with the project.

## FUNDING INFORMATION

This research was supported by an Australian Government Research Training Program Scholarship (2018–2020) and Andrews Meats Pty Limited (2020). Andrews Meats Industries provided funding to support the employment of two research assistants to assist in the collection of plate waste data and professional transcription of interviews. Ready‐made meal products were also provided by the company free of charge. Andrews Meats Industries was not involved in the design of the study, development of research questions, data collection tools, data collection or analysis. A written report was provided to Andrews Meats at the conclusion of the project outlining specific feedback provided by participants on the products used throughout the trial.

## ETHICS STATEMENT

Ethical approval was granted by the University of Queensland Human Research Ethics Committee (2020002082). Written informed consent was obtained, and verbal consent confirmed prior to interviews.

## CONFLICT OF INTEREST STATEMENT

The authors declare that there is no conflict of interest.

## Supporting information


**Data S1.** Supporting information.

## Data Availability

Research data are not shared.
