# Supplementary material for: Choice on the menu in residential aged care: An underrated tool for maintaining resident autonomy
Source: Nutr Diet. 2025 Feb 25;82(3):327–36. doi: 10.1111/1747-0080.70002 (PMC12168056; doi:10.1111/1747-0080.70002)
Supplement: Supplementary file 1 — Data S1. Supporting information. [file NDI-82-327-s001.docx]

**INTERVIEW GUIDE- Pre-Intervention**

**Interview Questions**

1. Do you have a favourite food? Is this served on the menu?
2. What do you think should happen at mealtimes?
3. What do you think it means to have a choice at mealtimes?
4. Do you think having a choice of meals is important? Prompt Why?
5. Do you feel like you have enough opportunities to makes choices about the food you eat?
6. How many choices do you think is needed?
7. Which meal would you like more choices at?
8. What happens if you do not like the meal?
9. Do you have input into the types of meals on the menu?
10. Do you feel your opinions or complaints are listened to?
11. Do staff support you to make choices? Prompt: How so?
12. How has the food in aged care been compared to your expectations?
13. If you could change one thing about the food, what would it be?
14. Do you have anything else you would like to say about the food and meals

**INTERVIEW GUIDE- Post-Intervention**

Interview Questions

1. What do you think about the new meals/system?
2. Is there anything you don’t like about the new system?
3. How do you feel about the food since the change?
4. What do you like most about the mealtime?
5. What do you like least about the mealtime?
6. Do you have enough choice (six options) with the new menu?
7. What are your thoughts about ordering your meals at the mealtime?
8. What do you think about the timing of the meals?
9. How has the new system impacted opportunities for you to make choices about your food?
10. From the previous interviews, residents commented on the lack of variety or options on the menu. Has that improved?
11. How has having more choice impacted you, do you think?
12. Do you like having choice at both lunch and dinner?
13. Is there anything you would like to see change?
